# Effect of Essential Oils and Dried Herbs on the Shelf Life of Fresh Goat Lump Cheese

**DOI:** 10.3390/foods13132016

**Published:** 2024-06-26

**Authors:** Miroslava Kačániová, Patrícia Joanidis, Jana Lakatošová, Simona Kunová, Lucia Benešová, Khurshed Ikromi, Farkhod Akhmedov, Khayyol Boboev, Mirzozoda Gulmahmad, Fariza Niyatbekzoda, Nasimjon Toshkhodjaev, Farkhod Bobokalonov, Nasimdzhon Kamolov, Natália Čmiková

**Affiliations:** 1 Faculty of Horticulture and Landscape Engineering, Institute of Horticulture, Slovak University of Agriculture in Nitra, Tr. A. Hlinku 2, 949 76 Nitra, Slovakia; n.cmikova@gmail.com; 2 School of Medical and Health Sciences, University of Economics and Human Sciences in Warsaw, Okopowa 59, 01043 Warszawa, Poland; 3 AgroBioTech Research Centre, Slovak University of Agriculture in Nitra, Tr. A. Hlinku 2, 949 76 Nitra, Slovakia; patricia.joanidis@uniag.sk (P.J.); jana.lakatosova@uniag.sk (J.L.); lucia.benesova@uniag.sk (L.B.); 4 Faculty of Biotechnology and Food Sciences, Institute of Food Sciences, Slovak University of Agriculture in Nitra, Tr. A. Hlinku 2, 949 76 Nitra, Slovakia; simona.kunova@uniag.sk; 5 Department of Food Production Technology, Technological University of Tajikistan, 63/3, N. Karabaeva Str., Dushanbe 734061, Tajikistan; x_teshaev@yahoo.com (K.I.); int.affairs.dep@gmail.com (F.A.); khbobo@mail.ru (K.B.); sosonholov@gmail.com (M.G.); lalbekfariza@gmail.com (F.N.); 6 Department of Food Technology, Khujand Polytechnic Institute of Tajik Technical University (KPITTU), 226, I. Somoni Avenue, Khujand 735700, Tajikistan; tnah@mail.ru (N.T.); farhodbobokalonov1331@gmail.com (F.B.); kamolovvet@mail.ru (N.K.)

**Keywords:** food preservation, storage period, antimicrobial activity, microbiological quality, microbiota of goat cheese

## Abstract

In recent years, the use of natural preservatives in food products has gained significant attention due to their potential health benefits and effectiveness. A standardized microbiological analysis was conducted on Slovak farm-produced lump goat cheese samples to determine the antibacterial activity of dry herbs and essential oils added to vacuum-packed goat cheese. We employed five dried herbs and five essential oils derived from the same plants. The microbiological quality of 145 fresh and vacuum-packed goat cheese samples was assessed. The number of coliform bacteria, total viable count, lactic acid bacteria, and microscopic filamentous fungi were examined in raw cheese samples stored for 12 days at 4 °C. All cheese samples were vacuum-packed (control samples were packed without vacuum). This study evaluated the potential benefits of using essential oils and dried herbs from thyme (*Thymus serpyllum* L.), black pepper (*Piper nigrum* L.), clove (*Eugenia caryophyllus* Thunb.), mint (*Mentha* × *piperita* L.), and basil (*Ocimum basilicum* L.) as preservatives. The essential oils were obtained from Hanus Ltd., Nitra, Slovakia, and were applied at a concentration of 2%. The dried herbs were obtained from Popradský čaj (Poprad, Slovakia) and Mäspoma Ltd. (Zvolen, Slovakia). The results showed that all microorganism groups were significantly reduced in cheese samples following the application of essential oils throughout the entire storage period. During the preservation of cheese samples in polyethylene bags used for vacuum packing food, *Lactococcus garvieae*, *L. lactis*, *Enterobacter cloacae*, and *Serratia liquefaciens* were the most frequently isolated microbiota. Essential oils and dried herbs demonstrated antimicrobial potential during the storage of vacuum-packed goat cheese.

## 1. Introduction

The ancient process of creating goat cheese, coupled with its unique sensory qualities, has increased consumer interest in these products [1]. However, these goods provide an ideal setting for the growth of microorganisms that can cause serious health issues or financial loss [2]. Cheese production is a complex process that requires high standards of hygiene and technology at each stage, in addition to high-quality milk [3]. The milk must be free from harmful bacteria. Additionally, inappropriate milk can be identified by high concentrations of butyric acid bacteria, coliform bacteria, and psychrotrophic microorganisms. Potentially harmful bacteria linked to raw goat milk include *Salmonella*, *Escherichia coli*, *Staphylococcus aureus*, and *Clostridium perfringens*, necessitating the pasteurization of the milk [4]. The microbial ecology of raw milk and the production environment, particularly for raw goat milk cheeses, significantly influence the sensory and nutritional qualities of the cheese [5,6,7]. Numerous studies have shown that the natural raw milk microbiota, primarily composed of *Lactobacillus* spp., has the ability to prevent the growth of foodborne pathogens during the cheesemaking process, ensuring microbial safety [8,9]. However, extensive research has indicated that utilizing endogenous milk microbial activity in preservation techniques may carry certain health hazards [10,11]. Foodborne outbreaks connected to handmade cheese consumption suggest a concern for sanitary authorities as a potential risk to public health as well as for artisanal producers as a potential damage to their brand identity [12].

Many naturally occurring spice components with specific antibacterial activities have been isolated. The use of active chemicals as food preservatives is not limited to in vitro investigations; food matrices are complex, and their physical and biological properties alter in real food systems [13]. Numerous studies have established that spices or their constituents can prevent food spoilage and serve as food preservatives. The antibacterial properties of spices in vitro have been well-documented over the past few decades, with microorganisms tested against extracts of whole plants or plant parts using various solvents [13]. Essential oils or active compounds, either alone or in combination, have also been tested for their action against several microorganisms [13,14,15]. These results indicate that spices have a broad range of antimicrobial activity against molds, yeasts, and both Gram-positive and Gram-negative bacteria [16].

The hydrosols of wild thyme, mint, sage, black pepper, and garlic, which are commonly used as food additives and drinks, were examined for their antibacterial properties against *Salmonella enteritidis* and *Bacillus subtilis*. The antibacterial activity of the previously utilized common herbs was assessed in vitro against these harmful bacteria using both single and combination hydrosols [17]. Many spices, including mint, clove, black pepper, basil, and wild thyme, demonstrated strong antibacterial and antifungal properties against pathogens, bacteria that cause food spoilage, *Bacillus subtilis* and *Pseudomonas fluorescens*, harmful fungi, *Aspergillus flavus*, and even microorganisms that are resistant to antibiotics, like methicillin-resistant *Staphylococcus aureus*. Spices, therefore, provide significant promise for the development of novel, secure antibacterial agents [18].

According to Alves-Silva et al. [19], essential oils derived from basil exhibit antimicrobial activity against various yeasts, including *Yarrowia lipolytica*, *Saccharomyces cerevisiae*, *Candida zeylanoides*, *Debaryomyces hansenii*, and *Pichia carsonii*, as well as bacteria such as *Shewanella putrefaciens*, *Achromobacter denitrificans*, *Enterobacter amnigenus*, *E. gergoviae*, and *Alcaligenes faecalis*. They also demonstrated the ability to suppress microscopic filamentous fungi like *Penicillium chrysogenum* and *Mucor racemosus*. Despite their antimicrobial activity varying depending on the spice type (origin and bioactive components), different bacteria respond differently to spices and herbs [20]. For instance, oregano essential oil exhibited stronger antibacterial action against *Listeria monocytogenes* compared to *Escherichia coli* [21]. Basil essential oils were effective against both *Bacillus subtilis* and *Staphylococcus aureus* [22]. Angelica root essential oil showed efficacy against a range of pathogens, including *E. coli* and *Bacteroides fragilis*, but was less effective against *Clostridium difficile*, *C. perfringens*, *Enterococcus faecalis*, *Eubacterium limosum*, and *Peptostreptococcus anaerobius* [23]. *Salvia rosmarinus* Spenn. essential oil displayed potent antibacterial activity against *L. monocytogenes* and *S. aureus* compared to *E. coli* [24]. Antifungal properties of spices, essential oils, and extracts have also been reported [16]. For example, basil essential oils were effective against *Candida albicans* [22].

The use of these preservatives has been evaluated in a variety of foods, including rice, fruit, dairy products, meat, fish, vegetables, and animal feed [16,25]. Hernández-Ochoa et al. [26] found that applying essential oils of clove and cumin to meat samples stored for 15 days at 2 °C reduced overall bacterial growth by 3.78 log CFU/g. The antibacterial activity of various spice extracts on raw chicken meat stored for 15 days at 4 °C was also investigated. Extracts of black mustard, clove, oregano, and cinnamon were effective against microbial growth when applied to raw chicken meat [27]. Essential oils of marjoram and coriander protected chickpea seeds by over 50% against *Aspergillus flavus* infestation [28]. Bay oil was found beneficial against *Alternaria alternata* infection in an in vivo study using cherry tomatoes [29]. In another trial, da Silveira et al. [30] used bay leaf essential oil to treat fresh Tuscan sausages, increasing the shelf life by two days and reducing the coliform population by 2.8 log CFU/g compared to untreated references. Rattanachaikunsopon and Phumkhachorn [31] treated fermented pork sausage with basil oil at 4 °C after inoculating it with *S. enteritidis*. After three days, bacteria in the basil oil were reduced from five to two log CFU/g, and sensory analysis indicated that these oil concentrations were suitable for human consumption [32]. Finally, Patrignani et al. [33] examined the use of spices in minimally processed fruits and vegetables and their ingredients.

Our hypothesis was to find out how the addition of dried herbs and essential oils affects the microbiological quality of fresh goat cheese during storage and whether their application has a significant effect on reducing or increasing different groups of microorganisms. The current study aimed to determine the shelf life of fresh vacuum-packed goat cheese refrigerated at 4 °C. The study assessed whether adding essential oils and dried herbs such as mint (*Mentha x piperita* L.), clove (*Eugenia caryophyllus* Thunb.), black pepper (*Piper nigrum* L.), basil (*Ocimum basilicum* L.), and wild thyme (*Thymus serpyllum* L.) could provide natural antibacterial treatment. Over a 12-day storage period, the investigation included monitoring of quantitative and qualitative microbiological changes at predetermined intervals.

## 2. Materials and Methods

### 2.1. Chemical Characterization of EO Samples via Gas Chromatography/Mass Spectrometry (GC/MS) and Gas Chromatography (GC-FID)

The chemical composition of the essential oils used has already been published in previous studies (Table 1) [34,35,36,37,38]. Gas chromatography/mass spectrometry analyses of the selected EO samples were performed using an Agilent 6890N gas chromatograph (Agilent Technologies, Santa Clara, CA, USA) coupled with a quadrupole mass spectrometer 5975B (Agilent Technologies, Santa Clara, CA, USA). An HP-5MS capillary column (30 m × 0.25 mm × 0.25 m) was used. The temperature program was as follows: 60 °C to 150 °C (increasing rate of 3 °C/min) and 150 °C to 280 °C (increasing rate of 5 °C/min). The total run time was 60 min. Helium 5.0 was used as the carrier gas with a flow rate of 1 mL/min. The injection volume was 1 L (EO samples were diluted in pentane), while the split/splitless injector temperature was set at 280 °C. The investigated samples were injected in the split mode with a split ratio of 40.8:1. Electron-impact mass spectrometric data (EI-MS; 70 eV) were acquired in scan mode over the *m*/*z* range 35–550. The mass spectrometry ion source and MS quadrupole temperatures were 230 °C and 150 °C, respectively. Acquisition of data started after a solvent delay time of 3 min. Gas chromatography (GC-FID) analyses were performed on an Agilent 6890 N gas chromatograph coupled with an FID detector. Column (HP-5MS) and chromatographic conditions were the same as for GC-MS. The FID detector temperature was set at 300 °C.

The individual volatile constituents of injected EO samples were identified based on their retention indices [39] and comparison with reference spectra (Wiley and NIST databases). The retention indices were experimentally determined using the standard method [40], which included retention times of *n*-alkanes (C6–C34) injected under the same chromatographic conditions. The percentages of the identified compounds (amounts higher than 0.1%) were derived from their GC peak areas.

### 2.2. Preparation and Packaging of Goat Cheese Samples

Pasteurized goat cheese (made from pasteurized goat milk; the production process involves heating the milk to a certain temperature (63 °C) for a set period (30 min) and then cooling it rapidly) was used in this experiment and was purchased from a private farm located in Dolný Hričov, Slovakia (49°13′55″ N, 18°36′57″ E). The fresh goat cheese’s physicochemical properties were as follows: pH of 4.9, 2.2% salt content, 27% protein content, 37.7% moisture content, and 50 g/100 g fat content. A total of four thousand grams of goat cheese was procured and refrigerated before being sent to the microbiological laboratory. The goat cheese was allowed to drain at 18 °C. Next, a sterile knife was used to cut each goat cheese into sections of around 25 g, and each portion was weighed separately.

With the exception of the control group, 100 µL of 2% mint (*Mentha x piperita* L., Lamiaceae), clove (*Eugenia caryophyllus* Thunb., Myrtaceae), black pepper (*Piper nigrum* L., Piperaceae), basil (*Ocimum basilicum* L., Lamiaceae), and wild thyme (*Thymus serpyllum* L., Lamiaceae) essential oils (EOs) from Hanus Ltd., Nitra, Slovakia, were applied to each 25 g cheese sample. Furthermore, dry herbs (mint, clove, black pepper, basil, and wild thyme) were added in powdered form, similar to spices commonly purchased in commercial food products.

Steam distillation of flowering clematis yielded *Mentha x piperita* L. EO, whose principal constituents are menthol (40.1%), menthone (16.8%), and menthyl acetate (9.1%) [34]. This essential oil comes from Australia and is made via steam distillation of flowering twigs. *Eugenia caryophyllus* Thunb. EO was mostly composed of 82.4% eugenol and 14.0% (E)-caryophyllene [35]. It was prepared by steam-distilling fresh leaves from Madagascar cloves. When fruits were steam-distilled to create *Piper nigrum* L. EO, key ingredients were (E)-caryophyllene (25.9%), sabinene (14.5%), and limonene (12.1%) [36]. This essential oil comes from India and is produced by steam distillation of the berries. According to the manufacturer, *Thymus serpyllum* L. EO is produced via steam distillation of flowering clematis with main constituents thymol (18.8%), carvacrol (17.4%), o-cymene (15.4%), and geraniol (10.7%) [38]. The plants are grown in Austria, and the EO is produced via steam distillation of the flowering twigs. *Ocimum basilicum* L. EO is produced via steam distillation of fresh flowering twigs sourced from Vietnam, with main constituents being methyl chavicol (estragole) (88.6%), 1,8-cineole (4.2%), and α-trans-bergamotene (1.7%) [37].

All dried herbs (mint, thyme, cloves, black pepper, and basil) were subjected to microbiological testing to ensure their safety before being used on the cheese samples. The herbs were sourced from two different suppliers: Popradský čaj (Poprad, Slovakia) supplied the mint and thyme, while the cloves, black peppercorns, and basil were obtained from Mäspoma s.r.o. (Zvolen, Slovakia). In addition, the dried herbs were combined with sunflower oil, which was also subjected to microbiological testing and confirmed to be free of microorganisms, before being used with the cheese samples. This mixture of sunflower oil + dried herbs and sunflower oil + EO was prepared before adding it to the cheese samples.

Sunflower oil (98 mL) was combined with two grams of herbs/two mL of EO. The goat cheese samples were then immersed in the oil mixed with 2% herbs/EO for one hour. Subsequently, the samples were packed into polyethylene bags using a vacuum packer (Concept, Choceň, Czech Republic). Care was taken not to damage the cheese. They were then vacuum-packed (see Table 2). Each 25 g cheese sample was individually wrapped. Vacuum packing was used for control sample groups (CA), control sample groups with oil (CV), and sample groups containing dried herbs (DH) and EO (MPV, MPEOV, ECV, ECEOV, PNV, PNEOV, OBV, OBEOV, TSV, and TSEOV).

To maintain consistent environmental conditions during storage, all cheese samples were kept in a controlled environment with regulated humidity and minimal exposure to light. The storage facility was equipped with climate control mechanisms to ensure stable temperature and humidity levels throughout the experimental period. Humidity was monitored regularly using hygrometers, and adjustments were made as necessary to maintain optimal conditions for cheese preservation. Additionally, measures were taken to minimize exposure to light, as it can accelerate microbial growth and affect the quality of the cheese. Cheese samples were stored in opaque containers or wrapped in light-blocking materials to prevent light-induced degradation. These stringent environmental controls were implemented to minimize external influences on the microbiological outcomes and ensure the integrity of the experimental results.

In the methodology, we implemented stringent quality control measures, including sterilization of equipment, microbiological testing of herbs and EOs, and meticulous handling procedures, to ensure sample integrity and prevent contamination.

### 2.3. Cultivation of Samples

The microbiological evaluations were carried out on the 0th (before treatment and packaging), 1st, 4th, 8th, and 12th days of storage at 4 °C. Goat lump cheese samples weighing twenty-five grams were then put into an aseptic stomacher bag. Subsequently, the samples were diluted to 10^−1^ using 225 mL of peptone water (Sigma-Aldrich, St. Louis, MO, USA), and they were homogenized for two minutes using a Stomacher. Pipetting an aliquot of 0.1 mL from the suitable dilution, standard pre-dried plate count agar medium was spread out.

For thirty minutes, the homogenized samples were shaken in a shaker (GFL 3031, Burgwedel, Germany). Plate count agar (PCA, Oxoid, Basingstoke, UK) was used to calculate total viable count (TVC) [41]. Inoculated Petri plates were then cultured for 48–72 h at 30 °C. Violet Red Bile lactose agar (VRBL, Oxoid, Basingstoke, UK) [42] was used to determine the presence of coliform bacteria (CB), and inoculated Petri plates were incubated at 37 °C for 24–48 h. Using Rogosa and Sharpe agar (MRS, Oxoid, Basingstoke, UK) and inoculated Petri dishes incubated with 5% CO_2_ at 30 °C for 48–72 h, lactic acid bacteria (LAB) [43] were counted. Using Potato Dextrose agar (PDA, Oxoid, Basingstoke, UK) [44], microscopic filamentous fungi (MFF) were measured. Inoculated Petri dishes were then cultured for 5–7 days at 25 °C. Three duplicates of each measurement analysis were performed. From each agar, eight different colonies were re-inoculated on Tryptone Soya agar (TSA, Oxoid, Basingstoke, UK) for the next identification by mass spectrometry.

### 2.4. Sample Preparation and Measurement Using MALDI-TOF MS

For the identification of isolated microorganisms from different media, MALDI-TOF MS was used. Using a Bruker MALDI-TOF (matrix-assisted laser desorption/ionization time of flight) MS Biotyper (Daltonics, Bremen, Germany), bacteria isolated from goat cheese samples were identified. Before identification, bacterial and yeast colonies were subcultured on Tryptone Soya Agar (TSA agar, Oxoid, UK) for 18–24 h. From each of the eight bacterial isolates, one colony was chosen. A mixture of 300 μL of distilled water (Sigma-Aldrich, St. Louis, MO, USA) and 900 μL of 99.8% ethanol (Bruker Daltonik, Bremen, Germany) was used to suspend the bacterial and yeast colonies. The mixture was then centrifuged for two minutes at 13,000 rpm. After removing the supernatant, the pellet was combined with 50 μL of 70% formic acid (*v*/*v*) (Sigma-Aldrich, St. Louis, MO, USA) and 50 μL of acetonitrile (Sigma-Aldrich, St. Louis, MO, USA). All chemicals used in this study were of the highest purity.

A steel plate was coated with 1 μL of the supernatant and allowed to air dry at 20 °C after another centrifugation. A further step involved applying 1 μL of MALDI matrix to the samples. The MALDI Biotyper 3.0 program (Bruker Daltonik, Germany) was used to evaluate the mass spectra data. The identification criteria were as follows: a score of 2.300 to 3.000 indicated a highly probable identification at the species level, a score of 2.000 to 2.299 indicated a secure genus identification with probable species identification, a score of 1.700 to 1.999 suggested probable identification at the genus level, and a score below 1.700 was considered unreliable for identification [45].

### 2.5. Statistical Analyses

Every measurement and analysis was performed in triplicate. The mean and standard deviation (SD) for the microbe count were determined using Microsoft Excel program 2311. A one-way ANOVA (main factor: treatment) was carried out using Prism 8.0.1 (GraphPad Software, San Diego, CA, USA). For post hoc analysis, Tukey’s test at α = 0.05 was conducted. SAS^®^ version 8 software was used to process the data.

## 3. Results

### 3.1. Microbiological Quality of Goat Cheese Samples in Different Days

**Figure 1:**
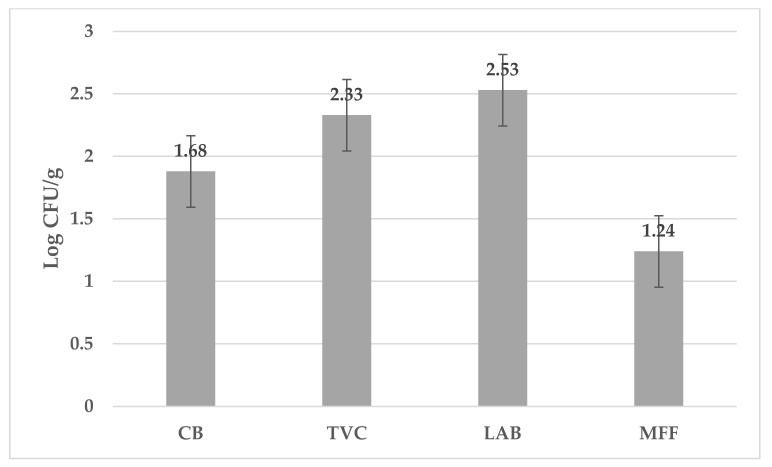
Counts (log CFU/g) of microorganisms in goat cheese samples during day 0 of storage. CB—coliforms bacteria, TVC—total viable count, LAB—lactic acid bacteria, MFF—microscopic filamentous fungi.

The microbiological quality of fresh goat lump cheese on day 0 is shown in Figure 1. The results present the number of coliform bacteria, total viable count, lactic acid bacteria, and microscopic filamentous fungi.

**Figure 2:**
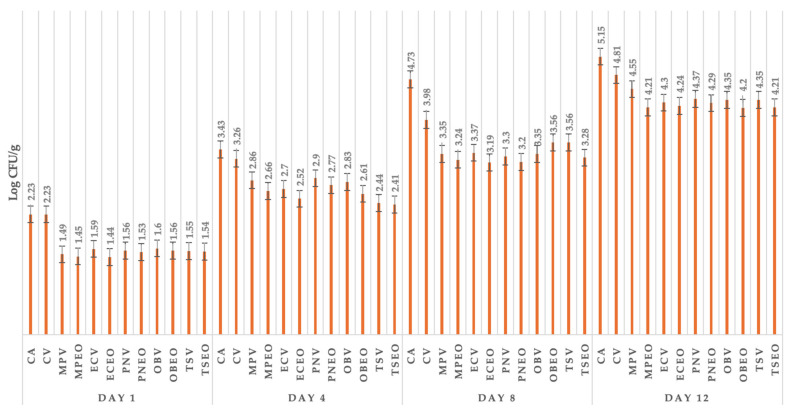
Coliform bacteria of goat lump cheese in all groups of samples stored from day 1 till 12. CA: control group in aerobic conditions; CV: vacuum-package control group; MPV: *Mentha x piperita* L. DH + vacuum; MPEOV: *Metha x piperita* L. EO + vacuum; ECV: *Eugenia caryophyllus* Thunb. DH + vacuum; ECEOV: *Eugenia caryophyllus* Thunb. EO + vacuum; PNV: *Piper nigrum* L. DH + vacuum; PNEOV: *Piper nigrum* L. EO +vacuum; OBV: *Ocimum basilicum* L. DH + vacuum; OBEOV: *Ocimum basilicum* L. EO + vacuum; TS: *Thymus serpyllum* L. DH + vacuum; TSEOV: *Thymus serpyllum* L. EO + vacuum.

Figure 2 shows the coliform bacteria of goat lump cheese from different groups of cheese samples, including control samples without or with packaging and those treated with EO and dried herbs (DH). The coliform bacteria on day 1 ranged between 1.44 log CFU/g in the group treated with *E. caryophyllus* Thunb. EO to 2.23 log CFU/g in the control group with aerobic storage conditions. The coliform bacteria on day 4 ranged from 2.41 log CFU/g in the group of cheese samples treated with *T. serpyllum* Thunb. EO to 3.43 log CFU/g in the control group with aerobic conditions. Similar results of coliform bacteria were found on day 8 as on the fourth day, with coliform bacteria present at the lowest levels compared to the control group in groups treated with EOs and DH, ranging from 3.19 log CFU/g in the group treated with *E. caryophyllus* Thunb. EO to 4.37 log CFU/g in the control group. The highest number of coliform bacteria was found on day 12, as in previous days, in the control group, with a count of 5.15 log CFU/g. On day 12, the most effective treatment against coliform bacteria was *O. basilicum* L. EO, with a value of 4.20 log CFU/g.

**Figure 3:**
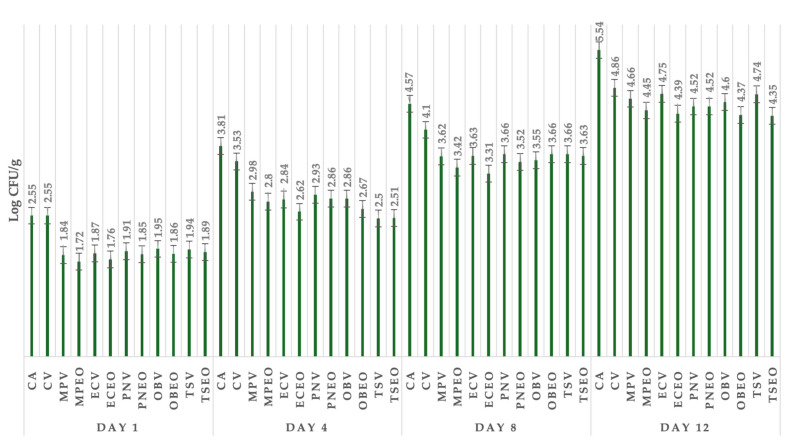
Total viable count of goat lump cheese in all groups of samples stored from day 1 till 12. CA: control group in aerobic conditions; CV: vacuum-packaged control group; MPV: *Mentha x piperita* L. DH + vacuum; MPEOV: *Metha x piperita* L. EO + vacuum; ECV: *Eugenia caryophyllus* Thunb. DH + vacuum; ECEOV: *Eugenia caryophyllus* Thunb. EO + vacuum; PNV: *Piper nigrum* L. DH + vacuum; PNEOV: *Piper nigrum* L. EO + vacuum; OBV: *Ocimum basilicum* L. DH + vacuum; OBEOV: *Ocimum basilicum* L. EO + vacuum; TS: *Thymus serpyllum* L. DH + vacuum; TSEOV: *Thymus serpyllum* L. EO + vacuum.

Figure 3 shows the total count of bacteria on all days. The total viable count on day 1 showed the best antimicrobial effect in the group with *M. piperita* L. EO, where the number of TVC was 1.72 log CFU/g, followed by the group treated with *E. caryophyllus* Thunb. EO. The most effective treatment against the total viable count on day 4 was *T. serpyllum* L. dried herbs, with a number of TVC of 2.50 log CFU/g. The most effective treatment against the total viable count was *E. caryophyllus* Thunb. EO, with a value of 3.31 log CFU/g. Total coliform bacteria ranged from 4.35 log CFU/g in the group treated with *T. serpyllum* L. EO to 5.54 log CFU/g in the control group.

**Figure 4:**
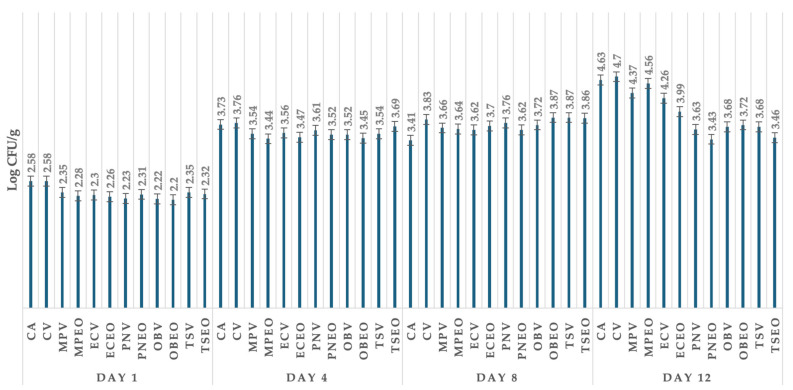
Lactic acid bacteria of goat lump cheese in all groups of samples stored from day 1 till 12. CA: control group in aerobic conditions; CV: vacuum-package control group; MPV: *Mentha piperita* L. DH + vacuum; MPEOV: *Metha piperita* L. EO + vacuum; ECV: *Eugenia caryophyllus* Thunb. DH + vacuum; ECEOV: *Eugenia caryophyllus* Thunb. EO + vacuum; PNV: *Piper nigrum* L. DH + vacuum; PNEOV: *Piper nigrum* L. EO + vacuum; OBV: *Ocimum basilicum* L. DH + vacuum; OBEOV: *Ocimum basilicum* L. EO + vacuum; TS: *Thymus serpyllum* L. DH + vacuum; TSEOV: *Thymus serpyllum* L. EO + vacuum.

Figure 4 shows lactic acid bacteria in goat lump cheese after 12 days. Lactic acid bacteria on the first day were highest in the control group without treatment (2.58 log CFU/g). In the control group on day 4 without treatment, the highest LAB was found, with a value of 3.73 log CFU/g. Lactic acid bacteria on day 8 ranged from 3.41 log CFU/g in the control group without treatment to 3.87 log CFU/g in the group of goat lump cheese treated with *O. basilicum* L. EO and wild thyme dried herbs. Lactic acid bacteria on day 12 were highest in the group of control samples packaged with vacuum and lowest in the group treated with *T. serpyllum* EO (3.46 log CFU/g).

**Figure 5:**
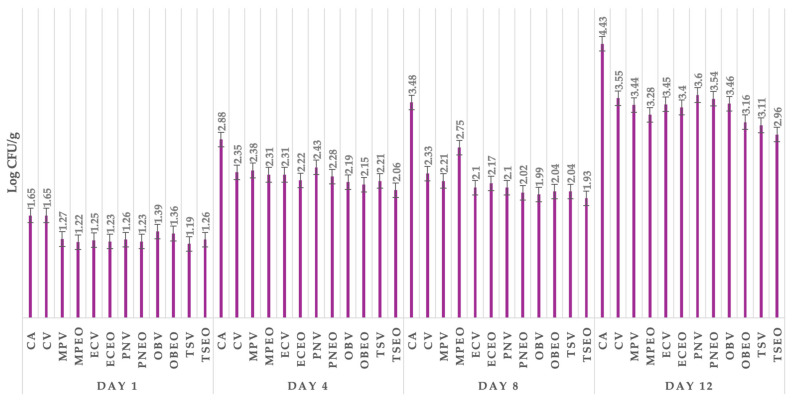
Lactic acid bacteria of goat lump cheese in all groups of samples stored from day 1 till 12. CA: control group in aerobic conditions; CV: vacuum-packaged control group; MPV: *Mentha x piperita* L. DH + vacuum; MPEOV: *Metha x piperita* L. EO + vacuum; ECV: *Eugenia caryophyllus* Thunb. DH + vacuum; ECEOV: *Eugenia caryophyllus* Thunb. EO + vacuum; PNV: *Piper nigrum* L. DH + vacuum; PNEOV: *Piper nigrum* L. EO + vacuum; OBV: *Ocimum basilicum* L. DH + vacuum; OBEOV: *Ocimum basilicum* L. EO + vacuum; TS: *Thymus serpyllum* L. DH + vacuum; TSEOV: *Thymus serpyllum* L. EO + vacuum.

All groups treated with EOs and dried herbs showed the lowest number of microscopic filamentous fungi, with the lowest number in the group treated with *T. serpyllum* L. dried herb (1.19 log CFU/g) combined with vacuum packaging on the first day (Figure 5). The lowest value of microscopic filamentous fungi was found in the cheese samples treated with *T. serpyllum* L. EO. Day 4 showed that the best antimicrobial potential was in the group treated with *T. serpyllum* L. EO and DH combined with vacuum packaging. The most visible occurrence of microscopic filamentous fungi on day 8 was found in the control group without any treatment. The most effective treatment against microscopic filamentous fungi was the group treated with *T. serpyllum* L. EO, with a value of 2.96 log CFU/g.

### 3.2. Isolated Species of Microorganisms

**Figure 6:**
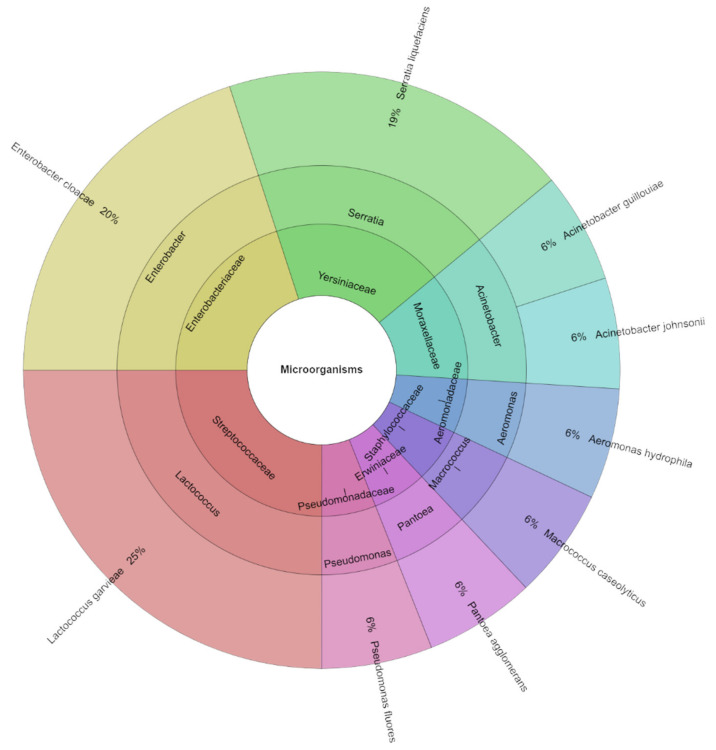
Krona chart: Isolated species, genera, and family from goat cheese on day 0.

On day 0, in the control group, before vacuum packaging and treatment with EO and dried herbs, a high count of 16 isolates was recorded. The most frequently isolated species were *Enterobacter cloacae*, occurring at 20%, followed by *Serratia liquefaciens* at 19% (Figure 6). In total, nine species, eight genera, and seven families were identified on day 0. The most commonly isolated families were Enterobacteriaceae and Yersiniaceae. These families belong to the domain Bacteria, order Enterobacterales in the class Gammaproteobacteria, and the phylum Pseudomonadota.

**Figure 7:**
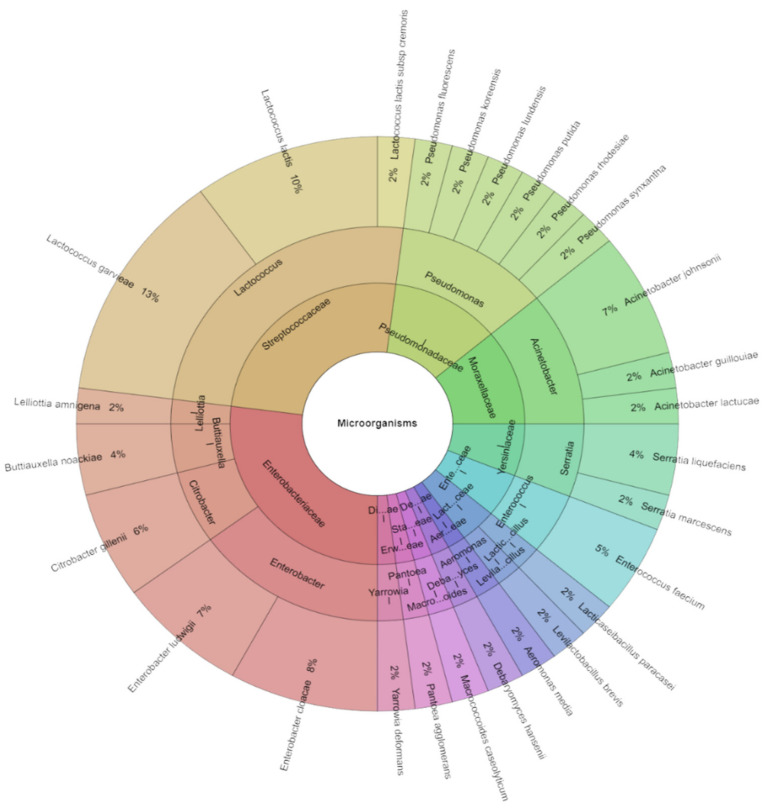
Krona chart: isolated species, genera, and family from goat cheese on day 1.

On day 1 (Table 3), a total of 27 species of microorganisms were found in both the control and experimental groups. A sum of 195 isolates was identified with high scores from lump goat cheese samples on the first day after storage. The most frequently isolated species across all groups were *Enterobacter cloacae* and *Lactococcus garvieae*, both found in 11 groups of samples. Other species like *Acinetobacter lactucea*, *Lacticaseibacillus paracasei*, *Lactococcus lactis* subsp. *cremoris*, *Macrococcoides caseolyticum*, *Pseudomonas fluorescens*, *P. korensis*, and *Yarrowia deformans* were isolated sporadically, each from only one group of tested samples. As previously mentioned, after the first day of storage, a total of 27 species of microorganisms, 16 genera, and 13 families (Figure 7) were isolated from all groups. Among these, the most frequently isolated species were *L. garvieae* (13%) and *L. lactis* (10%), followed *by E. cloacae* (8%), *E. ludwigii* (7%), and *A. johnsonii* (7%).

**Figure 8:**
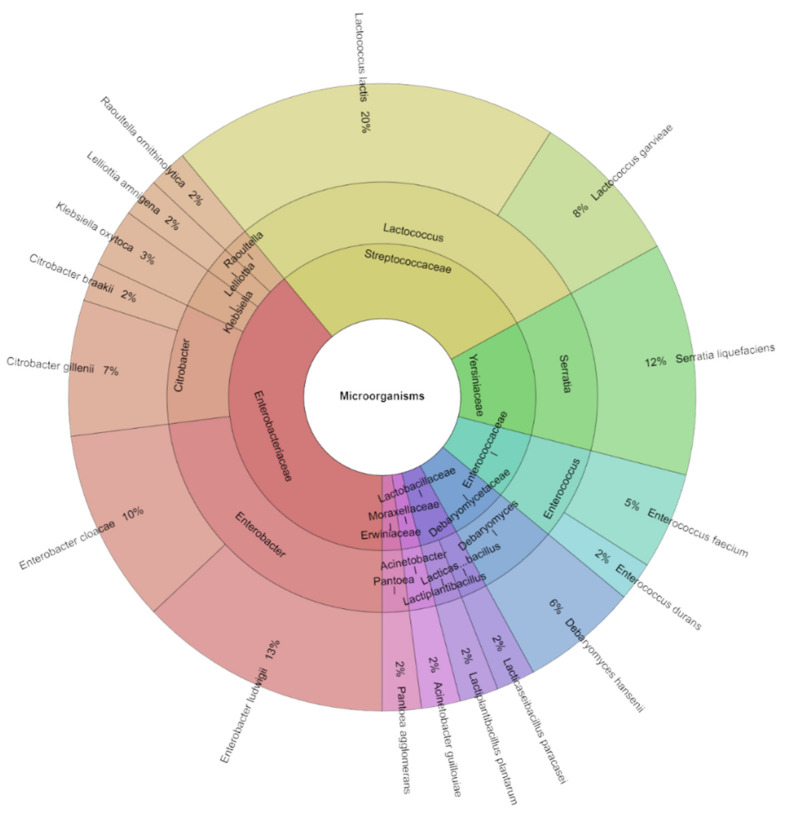
Krona chart: isolated species, genera, and family from goat cheese on day 4.

After 4 days of storage in lump goat cheese samples, a total of 17 species of microorganisms were found, with the most prevalent species across all groups being *L. lactis* (Table 4). A total of 194 isolates were identified with scores higher than 2.000. These isolated species of microorganisms belong to 13 genera and 8 families. Figure 8 illustrates that the most frequently isolated species were *L. lactis* (20%), followed by *E. ludwigii* (13%), *S. liquefaciens* (11%), *E. cloacae* (10%), and *L. garvieae* (8%). Comparing the results of day 1 and day 4, a lower number of isolates was found on day 4. Throughout both days, *Lactococcus* species were the most frequently isolated. This family is a member of the domain Bacteria, order Lactobacillales in the class Bacilli, and the phylum Bacillota.

**Figure 9:**
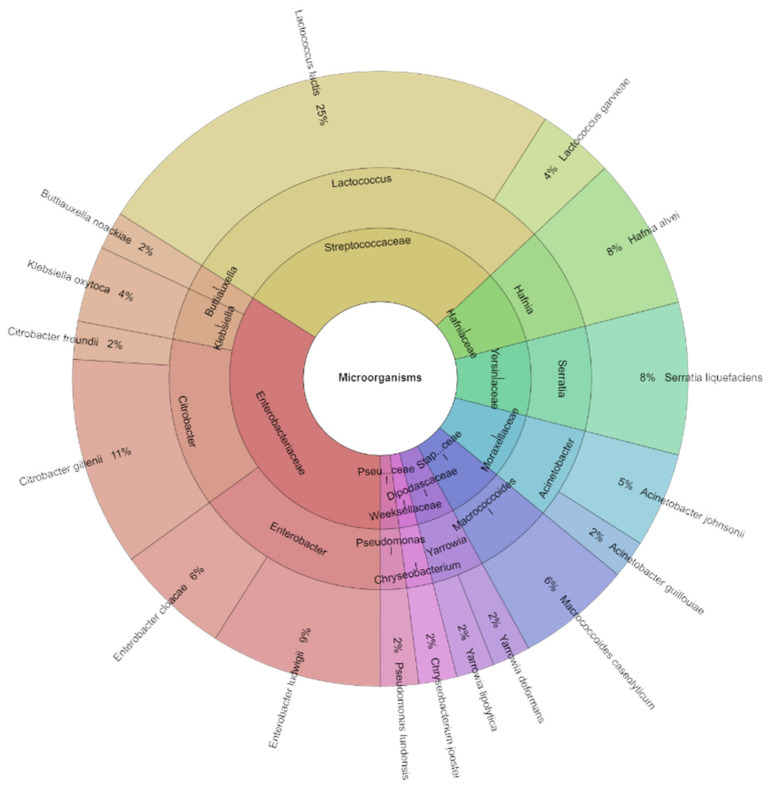
Krona chart: isolated species, genera, and family from goat cheese on day 8.

Table 5 displays the isolated and identified species presented in individual groups of goat cheese after 8 days of storage. Among all species, the most frequently occurring species across different groups was *L. lactis*, similar to the findings on the fourth day. In total, 8 families, 13 genera, and 17 species were isolated on day 8. A total of 193 isolates were identified using mass spectrometry. The most frequently isolated species on day 8 (Figure 9) was *L. lactis* (25%), followed by *Citrobacter gillenii* (11%) and *E. ludwigii* (9%).

**Figure 10:**
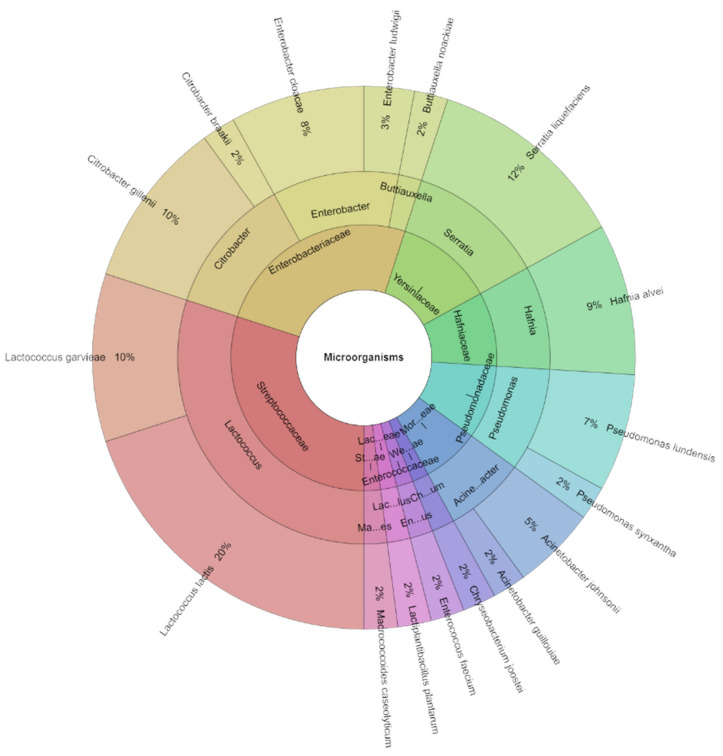
Krona chart: isolated species, genera, and family from goat cheese on day 12.

Table 6 displays the isolated species of microorganisms on day 12. *Citrobacter gillenii* was found in all groups of goat cheese. In total, 17 species, 12 genera, and 11 families were isolated on day 12. MALDI-TOF MS Biotyper identified 196 isolates with high scores. The most frequently isolated species from goat cheese on day 12 was *L. lactis* (Figure 10), accounting for 20% of the isolates. Other species with high percentages of isolation included *S. liquefaciens* (12%), *L. garvieae* (10%), and *C. gillenii* (10%).

## 4. Discussion

The microbial consortia present in milk and its byproducts are crucial bioindicators of animal health and play a significant role in the exchange of microorganisms between humans, animals, and the environment [46]. The technical process involved in cheese production, which includes developing unique flavors and aromas while ensuring the biosafety of dairy products, relies heavily on expertly orchestrating microbial metabolic processes [47]. Common cheeses are often made from raw, unprocessed milk, which harbors a high concentration of microbial diversity, the importance of which is still debated. On the one hand, using milk with a high microbial variety can aid in controlling food biosafety [48]. However, there is limited knowledge of how market storage conditions affect microbial processes during the shelf life of fresh goat milk cheese. Therefore, the focus of this investigation was on fresh goat milk cheese. We conducted the first study investigating the microbiota associated with traditional cheese made from raw goat milk, following the application of dry herbs and essential oils in vacuum-packed conditions for a 12-day shelf life.

In our study, commercially produced dried herbs were used for their antimicrobial effect, and these were added to individual samples of goat cheese during storage. Through microbiological analysis, we found that no microorganisms were present in the dried herb samples. In a different study, 96% of the inspected samples were of satisfactory or acceptable quality, and 90% of the samples were deemed “ready-to-use”. This study highlights the potential public health risks associated with adding spices and herbs to ready-to-eat foods that may not undergo additional processing. Effective decontamination and the use of hygienic growing, harvesting, and processing methods from farm to fork are essential for preventing microbial contamination in dried herbs and spices. Furthermore, it is imperative to follow proper food-handling procedures and ensure the correct consumption of herbs and spices by end consumers [49].

In our study, coliform bacteria ranged from 1.44 log CFU/g on the first day in the group of goat lump cheese treated with *Eugenia caryophyllus* Thunb. EO to 5.15 log CFU/g on the last day of storage in the untreated control group. Coliforms are commonly used as indicators of sanitary conditions in dairy facilities. However, there is still a limited understanding of the taxonomic diversity of coliforms in dairy environments. Coliforms have been detected in the microbial community of raw milk, according to several studies [50,51,52,53]. Yet, there are few studies on finished dairy products, such as raw goat milk cheese. In a different study, the maximum number of coliform bacteria was found to exceed the limit (2.4 × 10^7^ CFU/g) [54]. However, in our study, the coliform bacteria count in goat lump cheese remained within the limit. Coliform bacteria, including those belonging to the Enterobacteriaceae family, are considered crucial markers for the hygiene practices followed in manufacturing operations [55]. Specifically, enterobacteria have been found during cheese production and ripening processes and gradually decrease during maturation [56,57]. High levels of coliform contamination have also been reported in various cheeses made using traditional techniques from cow, goat, sheep, or mixed milk [58,59]. During the storage of cheese, *Origanum compactum* Benth. and *T. vulgaris* L. essential oils, at a concentration of 0.1%, demonstrated a noteworthy antimicrobial action on coliform bacteria. Coliforms have been completely absent from cheese aromatized with *O. compactum* Benth. EO on day 0 and from cheese aromatized with *T. vulgaris* L. EO on day 1 [60]. Our findings concur with those of Gammariello et al. [61], who reported that Thymus EO led to a minor reduction in coliforms.

The total viable count in our study ranged from 1.72 log CFU/g in the group of goat cheese samples treated with *Mentha x piperita* L. EO on the first day to 5.54 log CFU/g in the control group on the last day of the study. Another study found a higher total viable count in goat cheese samples [62]. Different results were reported by Mladenović et al. [63], where microbial loads ranged between 9.09 × 10^4^ CFU/g on day 0 and 1.24 × 10^8^ CFU/g on the 14th day. The number of lactic acid bacteria varied from 2.22 log CFU/g on the first day to 4.70 log CFU/g on the last day of storage. Lactic acid bacteria (LAB) are important for preventing infections and improving product quality in the dairy sector. Our findings contradict those of other studies, as we observed an increase in the number of LAB during storage in all tested groups. As predicted, the predominant microbiota of the cheese consisted of the four inoculated cultures’ microbial groups—thermophilic lactobacilli, thermophilic cocci, mesophilic lactobacilli, and enterococci—which increased from 7 to 9 log CFU/g on day 1 and remained constant until the end of the ripening period (30 days) [64]. The dairy sector often employs specific LAB cultures due to their advantages in microbiological safety, product homogeneity, and quality [65]. The population of microscopic filamentous fungi varied from 1.22 log CFU/g on the first day to 4.43 log CFU/g on the last day of the study. Only a small presence of yeast species was found in our study. In a different investigation, *Origanum compactum* Benth. essential oil outperformed *Thymus vulgaris* L. essential oil in terms of prolonging the shelf life of fresh goat cheese. Similar to our study, the overall aerobic microbiota was higher in this one [60]. Hamama et al. [66] have observed that LAB (10^8^–10^9^ CFU/g) dominated the soft goat cheese microbiota. All samples had a comparable total count of bacteria and lactic acid bacteria count, indicating that EOs had no effect on these bacterial populations. Our findings are consistent with those of Gammariello et al. [61], who showed that EOs had no effect on TCB and LAB. Furthermore, Lactic acid bacteria are the most resistant Gram-positive bacteria that are susceptible to EOs, as demonstrated by Conte et al. [67]. Our findings are consistent with the most recent research [68,69] that describes the effectiveness of EOs against yeasts and molds. Our findings similarly corroborate those of Gammariello et al. [61], who found that thymol increased an Italian goat cheese’s shelf life.

Several studies have examined the effects of essential oils and dried herbs on the microbiological quality of goat lump cheese during storage. Kunová et al. [45] found that dried oregano had the most potent inhibitory effect on the total viable count and coliform bacteria. Oregano EO was the most effective against fungi and yeasts. Zantar et al. [60] studied the impact of adding oregano and thyme EOs at concentrations of 0.05% and 0.1% on the microbiological characteristics of fresh ewe cheese stored at 8 °C. They found that both EOs fully suppressed the growth of coliforms from the first day of storage onward. Moreover, the addition of dried herbs and essential oils significantly improved the microbiological quality of the cheese compared to the control samples. Some authors have shown that dried herbs possess antibacterial properties [70,71]. EOs have been demonstrated to inhibit the growth of microscopic filamentous fungi or spoilage bacteria. Asensio et al. [72] showed that EOs significantly restricted yeast growth. EOs contain functional chemicals crucial for their antimicrobial activity, and several studies have demonstrated their high bactericidal effect [73]. Phenolic components can breach microbial membranes and enter cells, where they interfere with metabolic processes, exerting antibacterial activity. Many studies have reported a correlation between the chemical composition and anti-yeast properties of EOs [74].

A complicated regulatory environment encompassing quality requirements, ethical sourcing, labeling, and international trading applies to the essential oil manufacturing sector [75]. These rules aim to uphold the integrity of the sector, safeguard customers, and promote sustainability. Manufacturers face industry-specific challenges such as quality control, supply chain complexity, environmental impact, customer education, and competition, in addition to regulatory constraints [76]. Successfully navigating these obstacles calls for a thorough comprehension of the regulatory landscape, a dedication to quality, and a commitment to sustainable and moral business practices [77]. Manufacturers in this fast-paced, expanding industry who can rise to these challenges will prosper. Owing to their varied bioactive compositions, essential oils possess potent antibacterial and antioxidant characteristics, rendering them a perfect substitute for artificial food additives in commercial food items like goat cheese [78]. Today’s packaged food poses a serious risk to consumers of contracting food-borne illnesses because of artificial additives intended to preserve the organoleptic and microbiological qualities of food [79]. One natural remedy for these issues is to use the preservation qualities of essential oils. Essential oils serve as a natural flavoring agent in addition to extending the shelf life of products [80]. To reduce food-spoiling agents, essential oils, both alone and in combination, are also used in food packaging materials. The main goal of this research is to gain knowledge about the innovative uses of essential oils in the manufacturing of commercial food.

Using MALDI-TOF mass spectrophotometry, we further confirmed species allocation. The microbiota most frequently isolated in our investigation when cheese samples were preserved in bags included *Lactococcus garvieae*, *Lactobacillus lactis*, *Enterobacter cloacae*, and *Serratia liquefaciens*. Coliforms isolated from cheese samples were categorized into 13 genera in several studies. Among these genera were the environmental coliform genera *Hafnia*, *Raoultella*, and *Serratia*, which are the three most commonly isolated genera across all cheeses. The prevalence of *Escherichia*, *Hafnia*, and *Enterobacter* in raw milk cheeses was considerably higher [81]. Lactococci, pediococci, enterococci, and mesophilic lactobacilli are the main groups of lactic acid bacteria, as reported by Crow et al. [82]. Our findings are supported by other investigations such as those by Bettache and Mebrouk [83], Cheriguene et al. [84], and Tormo et al. [85]. Martín-Platero et al. [86] identified several species of Enterococcus in soft goat cheese and 36 species in hard goat cheese. Suzzi et al. [87] detected *Enterococcus faecalis*, *E. faecium*, *E. durans*, *E. hirae*, and *E. gallinarum* in goat smear-ripened cheese. Enterococci, specifically *E. faecalis* and *E. faecium*, were found in 63.6% of goat milk samples [88]. Psychrotrophic bacteria significantly influence cheese quality, with *Pseudomonas*, *Flavobacterium*, and *Alcaligenes* being the most commonly isolated genera from milk [54]. Although raw milk may also contain members of other genera [89,90], psychrotrophic yeasts and molds are also highly diverse. *Candida* spp., *Yarrowia lipolytica*, *Kluyveromyces marxianus*, *Geotrichum candidum*, *Debaryomyces hansenii*, and *Pichia* spp. are among the most frequently isolated microorganisms from goat cheese [91,92]. Species-level identification of bacteria is a crucial aspect of microbiology, and numerous publications have reported the successful identification of a wide range of bacteria using MALDI-TOF MS [93]. Our investigation confirmed the observed variation in identification between commercial identification technologies and MALDI-TOF MS at the genus level (57.2%) and species level (33.1%), as reported by Jesumirhewe et al. [93], Muruzović et al. [94], and Mladenović et al. [63]. Given its ability to provide more accurate identification, MALDI-TOF MS can potentially replace traditional biochemical approaches.

## 5. Conclusions

The rising popularity of goat milk cheese in the European Union underscores the importance of ensuring prolonged shelf life and maintaining high quality during long-term storage, especially for fresh lactic goat milk cheese, which is increasingly sought after by health-conscious consumers. Incorporating medicinal herbs and EOs directly into food packaging in specialized, non-reactive packs may prove beneficial for enhancing the longevity of various foods, including goat lump cheese. Our study demonstrated the antimicrobial potential of mint (*Mentha x piperita* L.), clove (*Eugenia caryophyllus* Thunb.), black pepper (*Piper nigrum* L.), basil (*Ocimum basilicum* L.), and wild thyme (*Thymus serpyllum* L.) when added to goat lump cheese as dried herbs and EOs. While modern supermarkets offer products with extended shelf life, the degree of shelf life extension achieved by incorporating herbs and EOs depends on various factors such as the type of food, spice, and preparation method. Further experimentation is necessary for precise determination in different food contexts.

## Figures and Tables

**Table 1:** Chemical composition of essential oils.

Compound	MPEO [34]	ECEO [35]	PNEO [36]	OBEO [37]	TSEO [38]
(*E*)-caryophyllene	2.2	14.0	25.9		
(*E*)-β-farnesene	0.4		0.2		
(*E*)-β-ocimene				0.4	
(*Z*)-β-ocimene			0.1		
1,8-cineole	5.20			4.2	1.5
1-octen-3-one					0.8
3-carvomenthenone	0.6				
3-octanol	0.3				
4-terpineol			2.1		1.8
bicyclogermacrene	0.3				
borneol					2.3
bornyl acetate					0.6
camphene			0.2		1.1
camphor				0.3	0.9
carvacrol					17.4
carvacrol methyl ether					0.5
carvone			0.1		
caryophyllene oxide		0.7	0.9		
*cis*-caryophyllene					2.4
cis-*p*-menth-2-en-1-ol			0.1		
*cis*-sabinene hydrate	1.5		0.5		
eugenol		82.4		0.3	
eugenol acetate		0.9			
geraniol					10.7
geranyl acetate					4.4
germacrene B			0.7		
germacrene D	1.7		0.3		
isomenthol	0.3				
isomenthone	2.8				
iso-menthyl acetate	0.4				
limonene			12.1		
linalool			0.4		5.3
linalool acetate					1.5
menthol	40.1				
menthone	16.8				
menthyl acetate	9.1				
methofuran	4.6				
methyl eugenol				0.2	
methyl chavicol				88.6	
*n*-amyl isovalerate	0.2				
neo-menthol	4.7				
ocimene	0.3				
*o*-cymene			1.2		15.4
*p*-cimene				0.7	
pulegone	1.5				
sabinene	0.5		14.5		
thymol					18.8
trans-sabinene hydrate			0.4		
trans-γ-bisabolene			0.2		
viridiflorol	0.2				
*α*-amorphene				0.2	
*α*-cis-bergamotene			0.2		
*α*-copaene			0.9		
*α*-guaiene			0.1		
*α*-humulene		1.8	2.0		1.0
*α*-limonene	1.8			0.4	1.2
*α*-phellandrene			1.2		
*α*-pinene	0.7			1.0	1.2
*α*-pinene			5.9		
*α*-selinene			1.0		
*α*-terpinene	0.3				1.1
*α*-terpinene			0.9		
*α*-terpineol			0.2		
*α*-terpinolene	0.,4			1.2	
*α*-terpinolene			0.7		
*α*-thujene			3.0		0.5
*α*-trans-bergamotene			0.2		
*α*-trans-bergamotene				1.7	
*β*-bisabolene			0.4		
*β*-bourbonene	0.5				
*β*-elemene			0.7		
*β*-myrcene	0.2				1.1
*β*-myrcene			1.6		
*β*-phellandrene			2.8		
*β*-pinene	1.10			0.2	0.3
*β*-pinene			7.6		
*β*-selinene			0.9		
*γ*-elemene			0.2		
*γ*-terpinene	0.5			0.4	8.1
*γ*-terpinene			1.4		
*δ*-3-carene			6.1		
*δ*-cadinene	0.5				
*δ*-cadinene			0.5		
*δ*-elemene			1.4		
Total	99.7	99.80	99.8	99.8	99.9

MPEO—*Mentha x piperita* L. EO; ECEO—*Eugenia caryophyllus* Thunb.; PNEO—*Piper nigrum* L.; OBEO—*Ocimum basilicum* L.; TSEO—*Thymus serpyllum* L. EO.

**Table 2:** The scheme of sample preparation.

Experimental Group	Indication	Samples Characteristic
Control group	CA	samples of goat cheese packaged under aerobic conditions and stored at temperature of 4 °C
Control group with vacuum packaging	CV	samples of goat cheese in vacuum packaging in polyethylene bags, stored at 4 °C
Experimental group with 2% *Mentha x piperita* L. dried herbs	MPV	samples of goat cheese treated with 2% of *M. piperita* L. dried herbs in vacuum packaging in polyethylene bags, stored at 4 °C
Experimental group with 2% *Mentha x piperita* L. essential oil	MPEOV	samples of goat cheese treated with 2% *M. piperita* L. EO in vacuum packaging in polyethylene bags, stored at 4 °C
Experimental group with 2% *Eugenia caryophyllus* Thunb. dried herbs	ECV	samples of goat cheese treated with 2% *E. caryophyllus* Thunb. dried herbs in vacuum packaging in polyethylene bags, stored at 4 °C
Experimental group with 2% *Eugenia caryophyllus* Thunb. essential oil	ECEOV	samples of goat cheese treated with 2% *E. caryophyllus* Thunb. EO in vacuum packaging in polyethylene bags, stored at 4 °C
Experimental group with 2% *Piper nigrum* L. dried herbs	PNV	samples of goat cheese treated with 2% *P. nigrum* L. dried herbs in vacuum packaging in polyethylene bags, stored at 4 °C
Experimental group with 2% *Piper nigrum* L. essential oil	PNEOV	samples of goat cheese treated with 2% *P. nigrum* L. EO in vacuum packaging in polyethylene bags, stored at 4 °C
Experimental group with 2% *Ocimum basilicum* L. dried herbs	OBV	samples of goat cheese treated with 2% *O. basilicum* L. dried herbs in vacuum packaging in polyethylene bags, stored at 4 °C
Experimental group with 2% *Ocimum basilicum* L. essential oil	OBV	samples of goat cheese treated with 2% *O. basilicum* L. EO in vacuum packaging in polyethylene bags, stored at 4 °C
Experimental group with 2% *Thymus serpyllum* L. dried herbs	TSV	samples of goat cheese treated with 2% *T. serpyllum* L. dried herbs in vacuum packaging in polyethylene bags, stored at 4 °C
Experimental group with 2% *Thymus serpyllum* L. essential oil	TSV	samples of goat cheese treated with 2% *T. serpyllum* L. EO in vacuum packaging in polyethylene bags, stored at 4 °C

**Table 3:** Presence of individual microbial species in control and experimental groups on day 1.

Species	CA	CV	MPV	MPEOV	ECV	ECEOV	PNV	PNEOV	OBV	OBEOV	TSV	TSEOV
*Acinetobacter guillouiae*			+			+			+		+	
*Acinetobacter johnsonii*	+	+		+	+		+	+		+		+
*Acinetobacter lactucae*	+											
*Aeromonas media*					+							
*Buttiauxella noackiae*		+	+				+			+	+	+
*Citrobacter gillenii*	+		+	+	+	+	+	+	+			+
*Debaryomyces hansenii*	+			+					+			
*Enterobacter cloacae*	+	+	+		+	+	+	+		+	+	+
*Enterobacter ludwigii*	+	+	+	+	+	+			+	+	+	+
*Enterococcus faecium*						+			+	+	+	+
*Lacticaseibacillus paracasei*												+
*Lactococcus garvieae*	+	+	+	+	+		+	+	+	+	+	+
*Lactococcus lactis*	+	+	+	+	+	+	+	+			+	
*Lactococcus lactis* subsp. *cremoris*										+		
*Lelliottia amnigena*						+		+		+		+
*Levilactobacillus brevis*						+				+		
*Macrococcoides caseolyticum*			+									
*Pantoea agglomerans*			+	+	+						+	
*Pseudomonas fluorescens*											+	
*Pseudomonas koreensis*		+										
*Pseudomonas lundensis*								+	+			
*Pseudomonas putida*			+					+				
*Pseudomonas rhodesiae*							+		+			
*Pseudomonas synxantha*					+							
*Serratia liquefaciens*			+	+	+	+			+	+	+	+
*Serratia marcescens*							+					
*Yarrowia deformans*										+		

CA: control group under aerobic conditions; CV: vacuum-packaged control group; MPV: *Mentha x piperita* L. DH + vacuum; MPEOV: *Metha piperita* EO + vacuum; ECV: *Eugenia caryophyllus* Thunb. DH + vacuum; ECEOV: *Eugenia caryophyllus* Thunb. EO + vacuum; PN: *Piper nigrum* L. DH + vacuum; PNEOV: *Piper nigrum* L. EO + vacuum; OBV: *Ocimum basilicum* L. DH + vacuum; OBEOV: *Ocimum basilicum* L. EO + vacuum; TS: *Thymus serpyllum* L. DH + vacuum; TSEOV: *Thymus serpyllum* L. EO + vacuum.

**Table 4:** Presence of individual microbial species in control and experimental groups on day 4.

Species	CA	CV	MPV	MPEOV	ECV	ECEOV	PNV	PNEOV	OBV	OBEOV	TSV	TSEOV
*Acinetobacter guillouiae*	+	+										
*Citrobacter braakii*								+	+			
*Citrobacter gillenii*	+			+	+	+	+		+	+	+	+
*Debaryomyces hansenii*		+	+					+	+	+		
*Enterobacter cloacae*	+	+		+		+	+		+	+	+	+
*Enterobacter ludwigii*	+	+	+	+	+	+	+	+	+	+		+
*Enterococcus durans*	+	+							+			
*Enterococcus faecium*	+	+	+		+	+		+			+	
*Klebsiella oxytoca*	+		+			+	+				+	
*Lacticaseibacillus paracasei*												
*Lactiplantibacillus plantarum*				+								
*Lactococcus garvieae*					+		+		+	+	+	+
*Lactococcus lactis*	+	+	+	+	+	+	+	+	+	+	+	+
*Lelliottia amnigena*									+			+
*Pantoea agglomerans*		+										
*Raoultella ornithinolytica*											+	
*Serratia liquefaciens*	+	+	+	+	+	+	+		+	+	+	+

CA: control group under aerobic conditions; CV: vacuum-packaged control group; MPV: *Mentha x piperita* L. DH + vacuum; MPEOV: *Metha piperita* EO + vacuum; ECV: *Eugenia caryophyllus* Thunb. DH + vacuum; ECEOV: *Eugenia caryophyllus* Thunb. EO + vacuum; PN: *Piper nigrum* DH + vacuum; PNEOV: *Piper nigrum* L. EO + vacuum; OBV: *Ocimum basilicum* L. DH + vacuum; OBEOV: *Ocimum basilicum* L. EO + vacuum; TS: *Thymus serpyllum* L. DH + vacuum; TSEOV: *Thymus serpyllum* L. EO + vacuum.

**Table 5:** Presence of individual microbial species in control and experimental groups on day 8.

Species	CA	CV	MPV	MPEOV	ECV	ECEOV	PNV	PNEOV	OBV	OBEOV	TSV	TSEOV
*Acinetobacter guillouiae*	+											
*Acinetobacter johnsonii*	+	+								+	+	+
*Buttiauxella noackiae*			+							+		
*Chryseobacterium joostei*												+
*Citrobacter freundii*							+					
*Citrobacter gillenii*	+	+	+	+	+	+	+	+		+	+	+
*Enterobacter cloacae*		+					+	+	+	+	+	+
*Enterobacter ludwigii*	+		+	+	+		+	+	+		+	+
*Hafnia alvei*		+	+	+	+	+	+	+	+	+		+
*Klebsiella oxytoca*			+	+		+	+		+			
*Lactococcus garvieae*			+			+		+				
*Lactococcus lactis*		+	+	+	+	+	+	+	+	+	+	+
*Macrococcoides caseolyticum*		+	+		+	+	+	+	+		+	+
*Pseudomonas lundensis*									+			
*Serratia liquefaciens*	+	+	+	+	+	+		+			+	
*Yarrowia deformans*	+				+							
*Yarrowia lipolytica*										+		

CA: control group under aerobic conditions; CV: vacuum-packaged control group; MPV: *Mentha x piperita* L. DH + vacuum; MPEOV: *Metha piperita* EO + vacuum; ECV: *Eugenia caryophyllus* Thunb. DH + vacuum; ECEOV: *Eugenia caryophyllus* Thunb. EO + vacuum; PN: *Piper nigrum* L. DH +vacuum; PNEOV: *Piper nigrum* L. EO + vacuum; OBV: *Ocimum basilicum* L. DH + vacuum; OBEOV: *Ocimum basilicum* L. EO + vacuum; TS: *Thymus serpyllum* L. DH + vacuum; TSEOV: *Thymus serpyllum* L. EO + vacuum.

**Table 6:** Presence of individual microbial species in control and experimental groups on day 12.

Species	CA	CV	MPV	MPEOV	ECV	ECEOV	PNV	PNEOV	OBV	OBEOV	TSV	TSEOV
*Acinetobacter guillouiae*	+							+				
*Acinetobacter johnsonii*	+	+		+		+	+					+
*Buttiauxella noackiae*		+								+		
*Chryseobacterium joostei*			+								+	
*Citrobacter braakii*		+					+					
*Citrobacter gillenii*	+	+	+	+	+	+	+	+	+	+	+	+
*Enterobacter cloacae*			+	+	+	+	+	+	+	+	+	+
*Enterobacter ludwigii*			+				+		+	+	+	
*Enterococcus faecium*	+											
*Hafnia alvei*	+	+	+	+	+		+	+	+	+	+	+
*Lactiplantibacillus plantarum*									+			
*Lactococcus garvieae*	+			+	+	+	+	+	+	+		+
*Lactococcus lactis*	+	+	+	+	+	+	+	+	+		+	+
*Pseudomonas lundensis*	+											
*Macrococcoides caseolyticum*			+	+		+	+	+	+	+	+	+
*Pseudomonas synxantha*										+		
*Serratia liquefaciens*	+	+	+	+	+	+		+	+	+	+	+

CA: control group under aerobic conditions; CV: vacuum-packaged control group; MPV: *Mentha x piperita* L. DH + vacuum; MPEOV: *Metha piperita* EO + vacuum; ECV: *Eugenia caryophyllus* Thunb. DH + vacuum; ECEOV: *Eugenia caryophyllus* Thunb. EO + vacuum; PN: *Piper nigrum* L. DH + vacuum; PNEOV: *Piper nigrum* L. EO + vacuum; OBV: *Ocimum basilicum* L. DH + vacuum; OBEOV: *Ocimum basilicum* L. EO + vacuum; TS: *Thymus serpyllum* L. DH + vacuum; TSEOV: *Thymus serpyllum* L. EO + vacuum.

## Data Availability

The original contributions presented in the study are included in the article, further inquiries can be directed to the corresponding author.
